# Fibre-Reinforced Composite for Protection against Shark Bites

**DOI:** 10.3390/ma13225065

**Published:** 2020-11-10

**Authors:** Thomas Fiedler, Trent Verstegen

**Affiliations:** School of Engineering, The University of Newcastle, Callaghan, NSW 2308, Australia; Trent.Verstegen@uon.edu.au

**Keywords:** shark protection, composite material, Kevlar fibers, UV exposure, seawater exposure, experimental testing

## Abstract

The number of shark attacks resulting in fatalities and severe injuries has increased steadily over recent years. This is mainly attributed to a growing population participating in ocean sports such as swimming, diving, and surfing. To mitigate the severity of shark attacks, the current study presents a novel fibre-reinforced composite for bite protection. This material is intended for integration into neoprene wetsuits, e.g., in the form of protective pads. A suitable material must be able to withstand significant bite forces, which are concentrated within a small contact area at the tips of the shark teeth. At the same time, the material should not hinder the complex motion sequences of aquatic sports. To this end, a novel fibre-reinforced composite was created by integrating Kevlar fibres into an elastic matrix. Uni-axial testing using shark teeth replicas was conducted on a specially designed test rig to quantify the effectiveness of the novel protective material.

## 1. Introduction

Despite its relatively small population, Australia is one of the world’s shark bite hotspots [1] with one of the highest numbers of human–shark interactions after the USA. Australian shark attack data has been recorded since 1791. To standardise reporting, the Australian Shark Attack File (ASAF) [2] was created in 1984 to update Australian shark attack cases and their outcomes. According to this data, three shark species are responsible for fatal attacks in Australia in the two decades following 1990: *Carcharodon carcharias* (great white shark), *Carcharhinus leucas* (bull shark), and *Galeocerdo cuvier* (tiger shark). During this time, *Carcharodon carcharias* was accountable for 68% of all unprovoked shark attack fatalities in Australia. Approximately half of these fatal attacks occurred during surface activities, mostly surfing (33%), whilst the remainder occurred whilst the victim was submerged, predominantly SCUBA diving (40%). Victims wearing a wetsuit account for 49% of all shark-attack cases, indicating that protective wetsuits may be an efficient measure to mitigate the severity of shark attacks. Since 1990, 139 injuries or fatalities were recorded and 61% of injuries occurred to the legs and 14% to the arms, whilst only 12% recorded bites to the torso. The recent rise in Australian shark attacks from an average of 6.5 per year (1990–2000), to 15 per year (2000–2010), and 22 per year since 2011 coincides with an increasing population and growing popularity for water-based activities [3]. A 2017 report by the Environment and Communications References Committee [4] recommended supporting the development of personal shark deterrent devices. The most common is an electromagnetic field generator ordinarily worn on an individual’s wrist or ankle; however, a recent study found that these devices showed limited effectiveness against *Carcharodon carcharias* [5]. Other approaches include the usage of sound to deter approaching sharks [6] and camouflage [7]. Another widely discussed strategy is the usage of surveillance drones [8] for the early detection of sharks to enable the pro-active evacuation of endangered ocean users. Drones seem particularly promising on patrolled beaches, but are unlikely to be widely adapted in remote locations. Somewhat more controversial is the suggestion to remove “problem individuals” from the shark population [9]. This approach is based on the assumption that fatal shark bites are linked to individual sharks rather than environmental factors or shark population densities.

Even the combined large-scale implementation of the technologies outlined above seems unlikely to eliminate the risk for future shark bites. Hence, there is a need for additional protection that can be used to complement the existing protective measures. Fibre-reinforced composites are commonly used in ballistic armour [10,11] and as protection against cutting injuries [12]. However, our preliminary tests indicated that these materials perform poorly when subjected to a sharp penetrant such as a shark tooth. Therefore, we propose a novel fibre-reinforced composite material to mitigate the severity of shark attacks. A similar approach was recently tested in an unrelated study [13]. These authors combined ultra-high molecular weight polyethylene fibres (UHMWPF) with neoprene to improve the protective properties of wetsuits. Uni-axial penetration testing using a single shark tooth was conducted, and peak forces prior to sample penetration were recorded. The integration of UHMWPF successfully increased puncture loads by a factor of approximately three compared to standard neoprene. It was further noted that the decreased flexibility of reinforced neoprene might negatively affect the wearer’s comfort and performance. Their comprehensive study further investigated the resistance of protective materials to sawing motions and in-vivo animal testing.

A comparison between ballistics damage and shark bites presents vastly different load cases; however, research into the composition and makeup of composites will carry between fields of research. Ballistic protection has long incorporated Kevlar composites that reduce trauma depth and absorb vast quantities of energy associated with projectile impacts. It was determined by Yavaş et al. [14] that trauma depth increases with a decreasing number of total layers in the composite samples tested. The energy absorption capability also decreases with a reduction in total number of layers. A decrease in energy absorption is explained due to the straining and fracture of yarns, delamination of plies and layers, friction energy between plies, and the mobility of yarns.

In the current study, we propose a Kevlar fibre reinforced composite embedded inside an elastic polymeric matrix. This is the first study to present this novel material and its degradation due to UV and seawater exposure. The design of this material poses a formidable optimisation challenge, balancing the best possible protection against the need for minimum weight and stiffness. Uni-axial penetration tests using shark teeth equivalents are performed in a controlled laboratory environment mimicking the loads of a shark bite. The tested samples contain five layers of plain weave Kevlar with an overall thickness ranging from 1.17–1.37 mm or eight layers from 1.92–1.98 mm. Prior to testing, selected samples were subjected to UV exposure and saltwater immersion to test for changes in the material’s performance.

## 2. Materials and Methods

### 2.1. Sample Preparation

**Figure 1:**
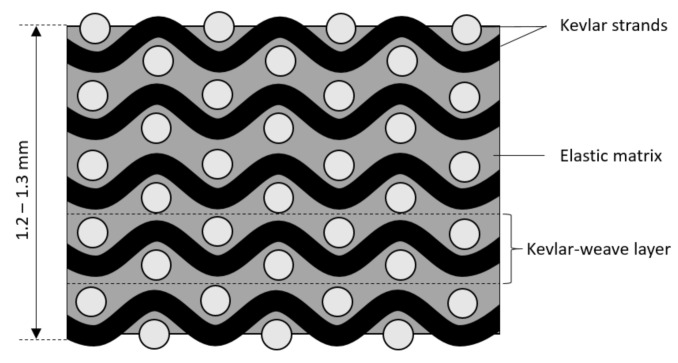
Schematic of the protective material.

All samples were provided by the company Aqua Armour (Newcastle, Australia). In brief, they were manufactured using plain weave Kevlar fibre mats obtained from Carbon Parts, QLD Australia. Preliminary research [15] indicated superior performance of this material against shark tooth penetration compared to Kevlar twill weave and other fibre types such as e-glass or carbon fibres. Five layers of woven Kevlar fibre (200 g/m^2^ plain weave, 9 warps and wefts/10 mm, thickness 0.26 mm) were stacked and infiltrated with a polyurethane based elastic polymer matrix. A schematic of the material is shown in Figure 1. In addition, one group of samples with eight Kevlar layers was produced. The matrix first protects the fibres against wear and environmental exposure. Second, it counteracts windowing of the fibres [16] where a sharp penetrator (such as a shark tooth tip) pushes the fibres aside before cutting them. The usage of an elastic matrix (as opposed to the more commonly used epoxy resins) decreases strength but creates a flexible material that can easily be bent by hand. This is an important requirement for their future incorporation into wetsuits where the wearer’s flexibility is paramount.

Nine different sample groups containing five samples each were produced. An overview of the tested samples is given in Table 1. It can be observed that seawater exposure caused a slight thickness increase of samples. All samples were of rectangular shape with the dimensions 90 mm × 90 mm. A single extended Kevlar layer with the dimensions 250 mm × 90 mm was integrated in each sample to facilitate sample fixation during penetration testing (see Section 2.2).

### 2.2. Testing Procedure

**Figure 2:**
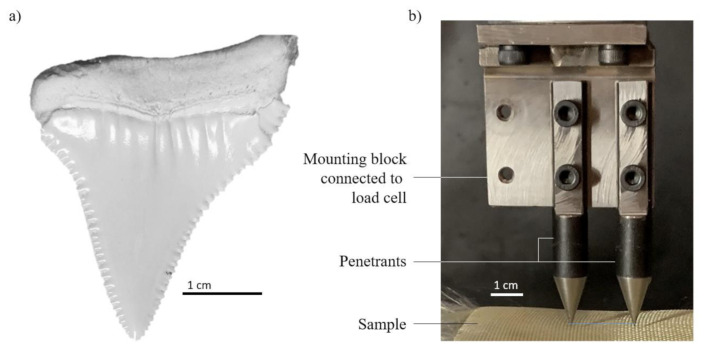
(**a**) *Carcharodon carcharias* tooth; (**b**) steel rod penetrants.

Initial penetration testing was conducted using real shark teeth [17]. However, the degeneration and fracture of the brittle teeth caused a lack of test repeatability and thus the transition to shark teeth replicas (see Figure 2b). These conical steel penetrants are topped by a 20° cone that approximates the geometry of the shark tooth tip. Due to their increased strength, no change of geometry was observed after repeated testing. Importantly, a sharp penetration tip was maintained during all tests; however, the serrated edges of shark teeth were not captured by the penetrants used. Two penetrants were mounted adjacent to each other mimicking the simultaneous engagement of two shark teeth. Their distance (21 mm) resembles the anatomy of a shark jaw and introduces possible interaction effects where the fabric is stretched between two contact points. The resulting multi-axial stress state may affect the deformation mechanisms of the material, such as windowing of the fibre strands. All penetration tests were conducted with a quasi-static machine-crosshead displacement velocity of 10 mm/min. Due to the relatively low deformation velocities occurring during a shark bite, no strain rate sensitivity effects are expected, and hence quasi-static results are considered representative.

For testing, samples were connected to a prismatic clay block with the dimensions 150 mm × 67 mm × ~40 mm. This was achieved by wrapping the single extended Kevlar layer around the block and then inserting the assembly into a slightly larger compression box, which was open towards the penetrant. The friction forces between the wrapped Kevlar layer and the clay proved sufficient to prevent significant sample motion. The intent of this approach was to simulate the integration of the protective material into a wetsuit surrounding a limb, where the clay replicates the resistance of human tissue. All tests were terminated after a penetrant displacement of 40 mm was reached to prevent contact between the penetrants and the lower surface of the compression box.

**Figure 3:**
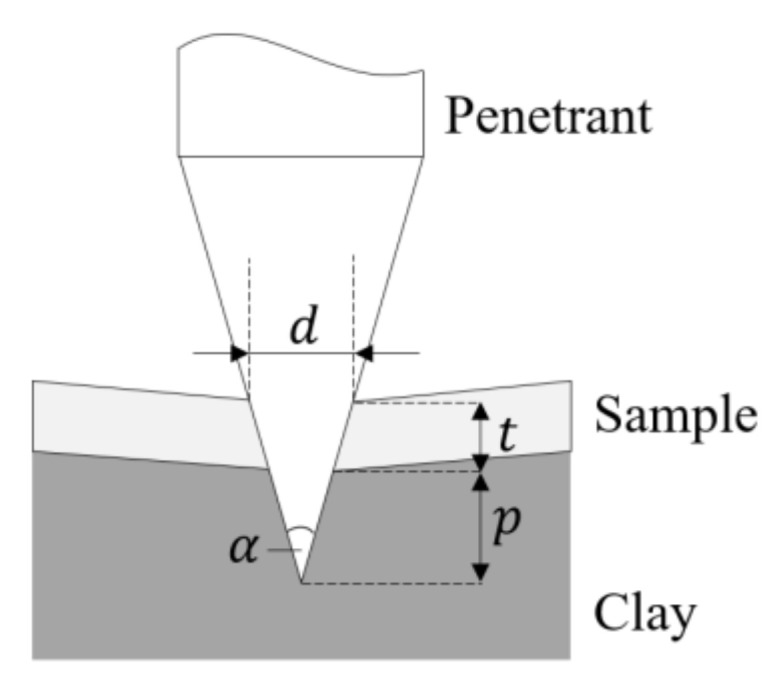
Calculation of the penetration depth *p*.

Tests were conducted on a 50 kN Shimadzu uni-axial testing machine (Shimadzu, Kyoto, Japan). The mounting block (see Figure 2b) was attached to the machine-crosshead and moved downwards towards the sample. During testing, the crosshead stroke s and the penetration force F  were measured. However, the stroke by itself is no useful measure for the effectiveness of the composite. The protective material is not designed to prevent deformation but instead to minimise penetration of the shark teeth beyond the material. The determination of this penetration depth p is visualised in Figure 3. After each 100 N load increment, tests were paused and the current penetrant diameter d was measured directly at the upper sample surface. This is a conservative approach where the diameter and thus the penetration depth may be slightly overestimated. Using Equation (1), the penetration depth p can then be calculated based on the sample thickness t and the penetrant cone angle $\alpha =20^{o}$.

(1) $$p=\frac{d}{2\cdot \tan\left(\frac{\alpha }{2}\right)}-t$$

After test completion and the removal of the sample, the calculated penetration depth of the final force was verified by a direct measurement of the depth of the conical imprint within the clay. Good agreement was obtained in all cases.

Prior to penetration testing, sample Groups 3–5 were exposed to oceanic saltwater. These exposure studies aimed to probe for possible performance changes resulting from aquatic use. Seawater was collected from Lake Macquarie, NSW Australia within a 5 L containment vessel. For sample Groups 4 and 5, the liquid was periodically replaced every 72 h. Following retrieval, samples were surface dried prior to penetration testing. Sample Group 5 was subjected to cyclic saltwater immersion and drying to replicate the usage pattern of a wetsuit. In total, 28 cycles were completed. During the night, samples were submerged in saltwater and dried in the sunlight during daytime. This caused additional UV exposure and the repeated formation of small salt crystals at the sample’s surfaces.

UV exposure is known to deteriorate the mechanical properties of Kevlar fibres [18]. All UV exposure samples were mounted on the north-facing roof of a domestic dwelling located at −32,980 S, 151,630 E from 19 May 2020. Sample Groups 6 and 7 were retrieved after 28 days and 130 days, respectively. It should be mentioned here that the selected exposure period coincides with the Australian winter/spring resulting in shorter days and reduced UV radiation exposure compared to the summer months.

Sample Group 8 was produced following the exposure tests outlined above. Samples were stored at room temperature for 28 days without direct UV exposure. The intent of this group was to quantify the strength increase due to the residual curing of the polymeric matrix.

The final sample Group 9 probes the change in penetration resistance when using three additional Kevlar weave layers, i.e., eight layers were used instead of five. These samples were manufactured alongside Group 8 and stored at the same conditions before testing after 28 days.

## 3. Results and Discussion

**Figure 4:**
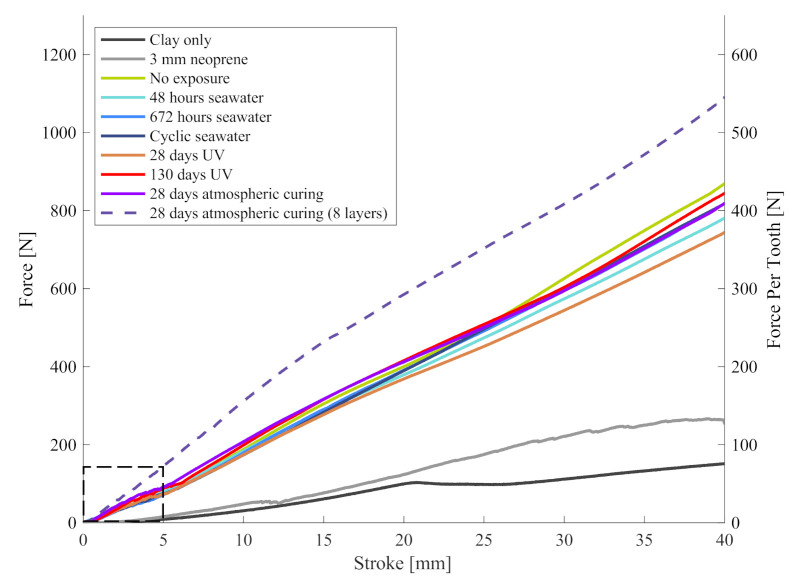
Force versus stroke.

Figure 4 shows the overall force (left *y*-axis) plotted against the penetrant stroke. The averaged force–stroke curves of each sample group are plotted and test groups (see Table 1) can be distinguished based on their line colour. The control tests (black) were conducted directly on the clay block without mounting any sample. Control tests were repeated periodically to ensure that penetrant and clay block did not change throughout the experiments. The same clay bock was reshaped and utilised for all experiments and no change in its properties was observed.

Group 1 tested standard neoprene wrapped around the clay bock thus mimicking the protection offered by conventional wetsuits. At low strokes below 20 mm, no significant difference between clay-only and neoprene samples is found. However, at higher strokes a force increase of approximately 50% is observed which may be explained by the elastic stretching of the neoprene. In [13], the puncture forces of protective materials have been measured to be between 400 N and 1150 N. Due to significant differences in the experimental setups (e.g., real shark tooth versus sharpened steel cone, different mounting of the sample, and dissimilar support materials) a direct comparison of these forces is invalid. As an example, the setup in [13] resulted in a 3 mm neoprene puncture force of 250–450 N. In contrast, we observed 3 mm neoprene penetration at tooth forces below 120 N. However, it may still be possible to compare force ratios of the protective material relative to the neoprene. Following this approach, we obtain an improvement factor of ~3.2 in the current study, which compares well to the ratio 3 observed in [13].

Group 2 (no exposure) exhibits the maximum forces at any given stroke (considering 5-layered samples). It is closely followed by Groups 4 and 5, which have been exposed to seawater for an extended time. Group 4 had been continuously submerged for 672 h, whereas Group 5 was removed and dried for 12 h each day before being reinserted, resulting in an effective submersion time of 336 h. The similar results of Groups 4 and 5 suggest that (i) no significant change occurs after 336 h immersion in seawater and (ii) repeated drying and formation salt crystals have no significant effect on the materials force–stroke characteristics. Interestingly, Group 3 (48 h seawater exposure) exhibits a slightly decreased load curve. UV exposure has been tested on sample Groups 6 and 7. The data suggests a decrease of the force after 28 days of UV exposure. However, samples exposed for 130 days show similar force–stroke curves to non-exposed samples (Group 2) and thus no conclusive trend can be derived. Overall, little deviation of the force–stroke data is observed for sample Groups 2–7, indicating a similar stiffness of these materials.

For comparison, Groups 8 and 9 were manufactured and cured at atmospheric conditions without exposure to sunlight or seawater. The force–stroke curve of Group 8 (five layers) closely resembles reference Group 2, even so a minor decrease of forces is observed. As expected, the addition of three supplementary Kevlar layers (Group 9) distinctly increases the force at any given stroke. This is partially attributed to the increased stiffness of these samples, which distributes the penetrant force over a larger surface area of the supporting clay.

**Figure 5:**
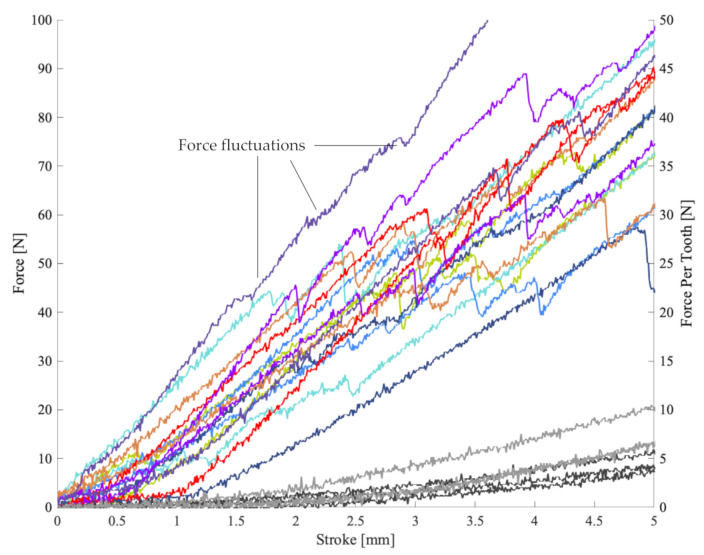
Magnified force–stroke curves.

A magnified view of the force–stroke curves in Figure 5 reveals distinct oscillations of the initial forces at strokes <5 mm. These fluctuations are attributed to the partial penetration of the Kevlar layers by the steel penetrants. The likely mechanism is windowing where the penetrant tip pierces through the gaps of the outermost Kevlar weaves. In some cases, multiple oscillations are visible which likely correspond to the initial penetration of individual Kevlar layers. As clearly visible in Figure 4, forces stabilise and increase steadily at a higher stroke. It appears that the elastic polymer matrix prevents further windowing and distributes the penetrant force over a larger sample section, thus activating the high strength of the Kevlar weaves.

**Figure 6:**
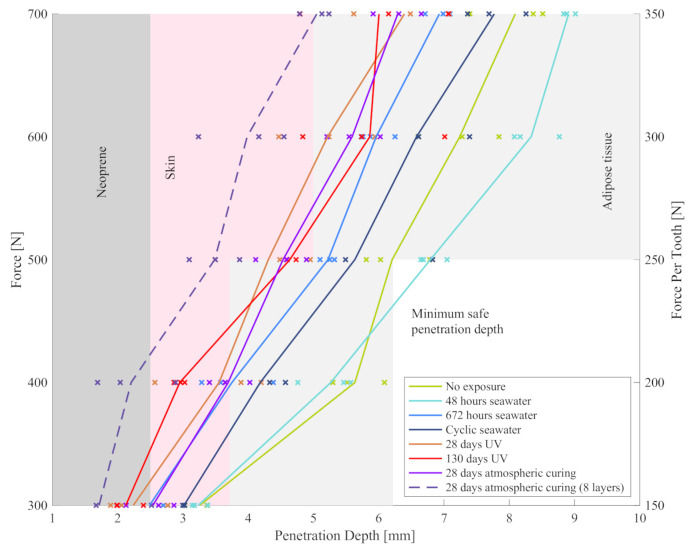
Force versus penetration depth.

High forces in Figure 4 are most likely related to an increased sample stiffness, which distributes the penetrant load over a larger section of the supporting clay. To better interpret these forces, it is important to remember that tests were aborted at a stroke of 40 mm to prevent collision between the penetrants and the metallic sample support. The reason for the large observed strokes was the deformation of the supporting clay that flowed away from the indentation site. If one assumes a similar behaviour of human soft tissue, sharks are unlikely to develop significant bite forces against such relatively soft targets. The 800 N to 4700 N bite forces of *Carcharodon carcharias* with 3.1 to 3.6 m length were measured using load cells [13]. These metallic devices exhibit a significantly higher stiffness than soft tissue, which enables the animals to develop higher bite forces. Similar bite forces have been predicted in numerical studies based on muscular and bone structure analysis of *Carcharodon carcharias* [19,20]. In addition, the exerted bite force will likely be distributed amongst multiple teeth and thus be spread over a larger surface area. This will reduce the effective bite force per tooth, which should be considered when interpreting Figure 4 and Figure 5. Nonetheless, high overall bite forces will likely result in severe compressive tissue injury and bone fracture. Such injury could be diminished by increasing the stiffness of the protective material; however, this would negatively affect mobility. It is thus important to note that the proposed composite will not protect effectively against such injuries. Instead, the key aim is to prevent the cutting of major arteries by shark teeth, a common cause of fatalities in shark attacks. This risk is addressed in Figure 6, which displays the force versus the penetration depth beyond the protective material.

The main protective mechanism of the novel composite is to limit penetration into the underlying tissue. If the protective material is mounted on a standard wetsuit with a neoprene thickness of 3 mm, tissue penetration will only commence after piercing the neoprene. During testing, the neoprene is compressed reducing its effective thickness to ~2.5 mm. This value was obtained by repeating selected tests with and without supporting neoprene and comparing the observed penetration depths into the clay. As a result, dermis penetration commences for some samples at a tooth force of ~150 N (see Groups 2 and 3). Surprisingly, apart from the eight-layer samples, the lowest penetration was observed for the UV exposure Groups 6 and 7, which on the other hand exhibited the lowest average penetration force at any given stroke (see Figure 4). Indeed, the highest penetration was observed for the non-exposed Group 2 and the 48 h seawater exposure Group 3. It is thus hypothesised that during UV exposure and, to a lesser degree, seawater immersion of the polymer matrix in the composite undergoes additional cross-linking resulting in improved penetration resistance. This effect clearly outweighs any possible performance reduction due to exposure. This theory is supported by good performance of sample Group 8 (28 days atmospheric curing) which resembles the UV groups. Increasing the number of Kevlar layers to eight (Group 9) distinctly decreases the penetration depth below 5 mm at the maximum load. In the case of neoprene (Group 1), penetration was measured to be 8.46 mm at 100 N and 12.28 mm at 150 N. Therefore, this data is not visible in Figure 6 and confirms that neoprene by itself is ineffective in preventing tissue penetration.

The penetration depths at 700 N range from 4.9 mm to 9.0 mm. It is important to interpret these values in the context of human anatomy. The average thickness of the human skin at the thigh has been measured to be in the range of 1.22 mm [21] to 1.97 mm [22] and is plotted as pink areas in Figure 6. Quantification of the adipose tissue layer thickness showed a strong variation between 1.48 mm to 31.6 mm with an average value of 7.92 mm (see grey areas). This adipose tissue data relates to patients suffering from diabetes and may deviate systematically for a population that engages regularly in aquatic sports. Hence, a conservative estimate of 2.5 mm is selected to represent vulnerable humans with thin adipose tissue layers. Assuming that major arteries are located predominantly underneath these layers, the acceptable penetration depth ranges from a minimum of 6.72 mm to an average of 12.89 mm (see Figure 6). Most cured samples satisfy the lower threshold and all tested samples fulfil the criterion based on the average values. Increasing the layer number to eight safely limits tissue penetration to non-critical depths. In summary, the protective material successfully limits penetration to a depth where damage of major arteries is unlikely to occur.

**Figure 7:**
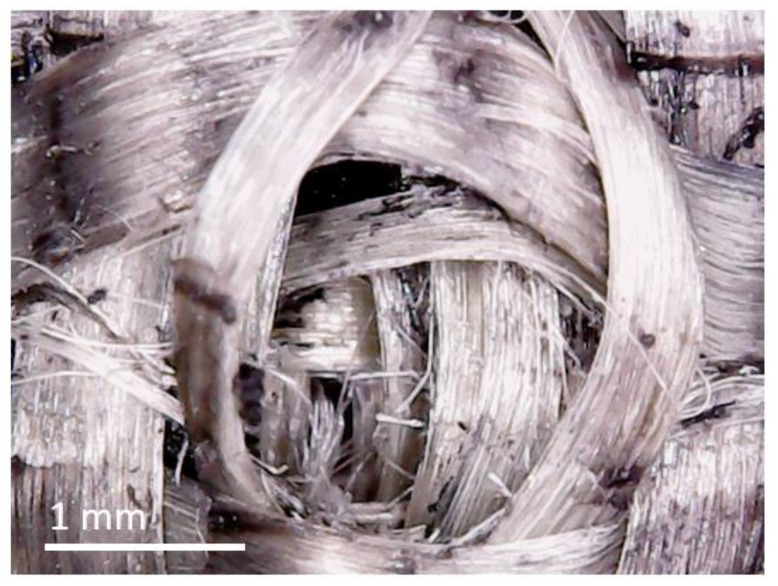
Magnification of penetration site.

Figure 7 shows a magnified photograph of the penetration testing site. The image was taken from a sample of Group 5; however, no significant difference between sample groups was observed. A small fraction of fibres have been cut, but windowing is clearly the predominant failure mechanism. Windowing is most pronounced in the topmost layer facing the penetrant. The image suggests that the polymeric matrix successfully suppresses windowing within the inner layers, thus counteracting penetration.

The current study demonstrates the feasibility of the proposed composite material to limit penetration into vulnerable tissue. However, the study has some inherent limitations, which will be addressed in our future research. Shark bites may involve complex motion sequences such as head shaking where the animal attempts to “saw” into its prey by activating the serrated tooth edges [13]. These are not captured in the current experimental setup. Furthermore, survivable shark attacks are often classified as ‘exploratory’ bites where the animal quickly withdraws after a bite to minimise its risk of injury from an unfamiliar prey [23,24]. It is thus unclear how animals will react when biting against the protective material. A previous study [13] had to utilise stimulants (sections of southern bluefin tuna) to entice the animals to bite on samples; however, this approach may have fundamentally altered the animal’s response. Testing samples with live animals still seems the best approach to obtain reliable data—accounting for the complexity of bite motion, momentum transfer, and shark behaviour—and will be pursued in our future research.

## 4. Conclusions

This paper introduced a flexible composite material for protection against shark bites. The tested material decreases the penetration of shark teeth replicas into vulnerable tissue and thus mitigates the severity of injury due to shark attacks. Several comparison studies were conducted to test for performance changes due to seawater and UV exposure. Based on the results, exposure had no significant impact on material performance. However, a desirable decrease of penetration depth was observed due to residual curing of the polymeric matrix of the composite. For cured samples, penetration of two sharpened steel rods resembling the teeth of *Carcharodon carcharias* was limited below 7 mm at 700 N compressive force. This penetration depth was further reduced to ~5 mm by including additional Kevlar layers into the protective materials.

## Figures and Tables

**Table 1:** Sample overview

Group	Description	Exposure Duration	Average Thickness Post Exposure (mm)
#1	Neoprene	N/A	3.00
#2	No exposure	N/A	1.25
#3	Continuous submersion in seawater	48 h	1.31
#4	Continuous submersion in seawater	672 h	1.33
#5	Cyclic submersion in seawater	28 × 12 h	1.31
#6	UV exposure	28 days	1.26
#7	UV exposure	130 days	1.20
#8	Cured at atmospheric conditions	28 days	1.23
#9	8 layers, cured at atm. conditions	28 days	1.95
